# Molecular Epidemiology and Clinical Features of Rotavirus Infection Among Pediatric Patients in East Java, Indonesia During 2015–2018: Dynamic Changes in Rotavirus Genotypes From Equine-Like G3 to Typical Human G1/G3

**DOI:** 10.3389/fmicb.2019.00940

**Published:** 2019-05-03

**Authors:** Alpha Fardah Athiyyah, Takako Utsumi, Rury Mega Wahyuni, Zayyin Dinana, Laura Navika Yamani, Soetjipto, Subijanto Marto Sudarmo, Reza Gunadi Ranuh, Andy Darma, Juniastuti, Dadik Raharjo, Chieko Matsui, Lin Deng, Takayuki Abe, Yen Hai Doan, Yoshiki Fujii, Hiroyuki Shimizu, Kazuhiko Katayama, Maria Inge Lusida, Ikuo Shoji

**Affiliations:** ^1^Indonesia-Japan Collaborative Research Center for Emerging and Re-emerging Infectious Diseases, Institute of Tropical Disease, Airlangga University, Surabaya, Indonesia; ^2^Department of Child Health, Soetomo Hospital, Airlangga University, Surabaya, Indonesia; ^3^Center for Infectious Diseases, Kobe University Graduate School of Medicine, Kobe, Japan; ^4^Department of Virology II, National Institute of Infectious Diseases, Tokyo, Japan; ^5^Laboratory of Viral Infection I, Department of Infection Control and Immunology, Kitasato Institute for Life Sciences and Graduate School of Infection Control Sciences, Kitasato University, Tokyo, Japan

**Keywords:** rotavirus, equine-like G3, typical human genotype, genotype change, clinical feature, Indonesia

## Abstract

Group A rotavirus (RVA) is the most important cause of severe gastroenteritis among children worldwide, and effective RVA vaccines have been introduced in many countries. Here we performed a molecular epidemiological analysis of RVA infection among pediatric patients in East Java, Indonesia, during 2015–2018. A total of 432 stool samples were collected from hospitalized pediatric patients with acute gastroenteritis. None of the patients in this cohort had been immunized with an RVA vaccine. The overall prevalence of RVA infection was 31.7% (137/432), and RVA infection was significantly more prevalent in the 6- to 11-month age group than in the other age groups (*P* < 0.05). Multiplex reverse transcription-PCR (RT-PCR) revealed that the most common G-P combination was equine-like G3P[8] (70.8%), followed by equine-like G3P[6] (12.4%), human G1P[8] (8.8%), G3P[6] (1.5%), and G1P[6] (0.7%). Interestingly, the equine-like strains were exclusively detected until May 2017, but in July 2017 they were completely replaced by a typical human genotype (G1 and G3), suggesting that the dynamic changes in RVA genotypes from equine-like G3 to typical human G1/G3 in Indonesia can occur even in the country with low RVA vaccine coverage rate. The mechanism of the dynamic changes in RVA genotypes needs to be explored. Infants and children with RVA-associated gastroenteritis presented more frequently with some dehydration, vomiting, and watery diarrhea, indicating a greater severity of RVA infection compared to those with non-RVA gastroenteritis. In conclusion, a dynamic change was found in the RVA genotype from equine-like G3 to a typical human genotype. Since severe cases of RVA infection were prevalent, especially in children aged 6 to 11 months or more generally in those less than 2 years old, RVA vaccination should be included in Indonesia’s national immunization program.

## Introduction

Acute gastroenteritis is a common and important cause of childhood morbidity and mortality in developing countries (Elliott, 2007). Diarrhea, the most common symptom of acute gastroenteritis, remains the third leading cause of overall morbidity and the leading cause of infant mortality in Indonesia, and 38–67% of children with diarrhea admitted to hospitals in Indonesia were infected with group A rotavirus (RVA) (Soenarto et al., 1981; Sudarmo et al., 2015; Nirwati et al., 2016). RVA, which is known as ‘winter diarrhea,’ exhibits distinct seasonality in temperate areas, whereas the effect of season on RVA infection is not extreme in the tropics (Levy et al., 2009).

RVA belongs to the *Reoviridae* family and is a non-enveloped virus, consisting of 11 segments of double-stranded RNA and encoding six structural and six non-structural proteins (NSPs). RVA is classified into 35 G and 50 P types on the basis of VP7 (glycoprotein) and VP4 (protease-sensitive) protein (Rojas et al., 2017), respectively. RVA genotypes G1P[8], G2P[4], G3P[8], G4P[8], G9P[8], and G12P[8] are commonly found worldwide (Santos and Hoshino, 2005; Dóró et al., 2014). These genotypes were also commonly detected in Indonesia during 2010–2015 (Sudarmo et al., 2015; Mulyani et al., 2018). There are also three genotype constellations of human RVA, termed Wa-like (G1-P[8]-I1-R1-C1-M1-A1-N1-T1-E1-H1), DS-1-like (G2-P[4]- I2- R2-C2-M2-A2-N2-T2-E2-H2) and the less common AU-1-like (G3-P[9]- I3- R3-C3-M3-A3-N3-T3-E3-H3) (Matthijnssens et al., 2008). The G1, G3, G4, G9 and G12 are usually grouped into Wa-like constellation, and G2 and G8 are grouped into DS-1-like constellation. We recently reported equine-like G3P[8]/P[6] strains with a DS-1 like backbone (equine-like G3-P[8]/[6]- I2- R2-C2-M2-A2-N2-T2-E2-H2), resulting from a rare human/equine reassortment, circulating among pediatric inpatients with acute gastroenteritis at a private hospital in the suburbs of Surabaya, Indonesia (Utsumi et al., 2018). All the tested samples were determined to be the equine-like G3 genotype, which was initially isolated in Australia and Japan, later emerging globally (Malasao et al., 2015; Arana et al., 2016; Cowley et al., 2016; Doro et al., 2016; Guerra et al., 2016; Komoto et al., 2018; Pietsch and Liebert, 2018). A report from Australia pointed out that equine-like G3P[8] was more common in the areas where Rotarix^®^ was being used (Roczo-Farkas et al., 2016), and another study found that the equine-like G3P[8] strain was responsible for the most episodes of severe gastroenteritis (Cowley et al., 2016).

Two kinds of RVA vaccines (Rotarix^®^ and RotaTeq^®^) were licensed in 2006, and 84 countries have introduced Rotarix^®^ or RotaTeq^®^ into their national immunization programs (Abou-Nader et al., 2018). The introduction of RVA vaccines into human populations may impose a selective pressure on circulating RVA strains especially in the post-vaccine era (Matthijnssens et al., 2010), although the current RVA vaccines are highly effective. Post-vaccination surveillance studies in the countries where the Rotarix^®^ vaccine was commonly used, showed significant reduction of RVA prevalences and a genotype shift in proportion from G1P[8] to G2P[4] strains (Dóró et al., 2014; Mukhopadhya et al., 2017). Indonesia has not included an RVA vaccine in its national immunization program yet, even though RVA vaccines have been commercially available since 2011. Little is known about the coverage and impact of RVA vaccines in Indonesia.

The purpose of the present study was to evaluate the transition of prevalences and genotypes of RVA and the severity of RVA-associated acute gastroenteritis among children admitted to a referral hospital in East Java, Indonesia.

## Materials and Methods

### Study Populations and Stool Samples

A total of 432 stool samples were collected from pediatric patients who were admitted to a government-owned hospital with a clinical diagnosis of acute gastroenteritis between September 2015 and March 2018. This hospital is a referral hospital in East Java, located in Surabaya, the second-largest city in Indonesia. The study population ranged in age from 1 month to 15 years and 11 months (median age, 12.5 months). The patients were classified into 7 age groups: <6, 6–11, 12–23, 24–35, 36–47, 48–59, and ≥60 months. The criterion for acute gastroenteritis was defined as the occurrence of ≥3 times watery or looser-than-normal stools per day and lasts for less than 14 days. To assess the shifting of RVA genotypes from 2013 to 2018 in East Java, 4 RVA-positive stool samples collected in 2013 and 3 in 2014 from pediatric patients with acute gastroenteritis at the same hospital were randomly included. None of the patients in this cohort had been immunized with an RVA vaccine. Demographic data (age, sex) and clinical features (type of stool, vomiting, diarrhea, fever, dehydration) were recorded. The severity of dehydration was determined using the WHO criteria (World Health Organization [WHO], 2014). The seasonality regional rainfall data of Surabaya were obtained from the ClimatView organized by Japan Meteorological Agency^1^, because rainfall has been considered as the indicator for seasonality in Tropic areas. Written informed consent was obtained from parents or guardians of all the children. The study protocol was reviewed and approved by the ethics committees of the government-owned hospital (No. 188) and Airlangga University (No. 2054) in Indonesia, and of Kobe University (No. 1857) in Japan.

### Rotavirus Detection by Immunochromatography Test

All the stool samples were screened for RVA antigen using the Dipstick “Eiken” Rota kit (Eiken Chemical, Co., Tokyo, Japan).

### RNA Extraction and RT-PCR Genotyping

Viral RNA was extracted from 140 μl of the supernatant with a QIAamp Viral RNA mini kit (Qiagen, Hilden, Germany). Positive samples determined to be positive by an immunochromatography test were subjected to genotyping in the VP7 (G typing) and VP4 (P typing) genes by multiplex reverse transcription-PCR (RT-PCR) with newly designed primers for equine-like G3 in the VP7 gene (Fujii et al., 2019b), and with the primers for VP4 that have been designed and used in our lab (Table 1). Equine-like G3 in the VP7 was typed with the primer G3e-757F (Table 1).

**Table 1:** Primer set for VP7 genotyping.

	Primer	5′-Sequence-3′	Position	Product size (bp)
1st PCR	VP7 C-040F	CTCCTTTTAATGTATGGTATTGAATATACC	40–69	
	VP7 C-941R	GTATAAAANACTTGCCACCATTTTTTCCA	913–941	902
2nd PCR	VP7 C-0932R	ACTTGCCACCATTTTTTCCA	913–932	
	G1-297F	GTATTATCCAACTGAAGCAAGTAC	297–320	636
	G2-401F	TTAAAGACTACAATGATATTACTACATT	401–428	532
	G3-809F	CAAGGGAAAACGTRGCAGTTA	809–829	124
	G3e-757F	CTAGATGTTACTACGGCTAC	757–776	176
	G4-478F	TTCGCTTCTGGTGAGGAGTTG	478–498	455
	G8-179F	TTACRCCATTTGTAAATTCACAG	179–201	754
	G9-606F	GATGGGACARTCTTGTACCATA	606–627	327
	G12-669F	TACRACAACCGACGTCACA	669–687	264

**Primer set for VP4 genotyping**		

1st PCR	VP4 C Ad-F	GGGGGCTATAAAATGGCTTC	1–17	
	VP4 C-819r	CTCTATTATATTGCATTTCTTTCCA	790–814	822
2nd PCR	VP4 C Ad-F	GGGGGCTATAAAATGGCTTC	1–17	
	P4-361r	GCCTATTTGTTTGACTRACATG	340–361	364
	P6-613r	CATGTATTACAGTTTCTACTTCAG	590–613	616
	P8-447r	CTGCYTCTAAACATTTCTAAAAAC	423–447	450
	P9-193r	GCACTAATGTAGAATCAGGCAA	172–193	196

*Some degenerate base symbols have been used (R = A or G, N = A, T, G or C).*

### Nucleotide Sequencing and Phylogenetic Analyses

Twenty-three (4 collected in 2013, 3 collected in 2014, and 16 collected in 2015–2018) RVA-positive specimens detected by RT-PCR contained sufficient RNA for further whole genome characterizations and were analyzed by next-generation sequencing (NGS). The cDNA library preparation and Illumina MiSeq (Illumina, San Diego, CA, United States) sequencing were performed as previously described (Doan et al., 2017). The full-length nucleotide sequence of each genome segment of the RVAs was obtained using CLC Genomics Workbench v7.0.3 software (CLC Bio, Aarhus, Denmark) with the assembled contigs as query sequences and 11 genome segments of a reference RVA as the target sequences. The genotypes of the 11 genome segments of the 23 Indonesian strains were determined using the RotaC v2.0 web-based classification tool^2^. The genome sequences were aligned with reference sequences using CLUSTAL X (version 1.83) software, and the phylogenetic trees were constructed by the neighbor-joining method. To confirm the reliability of the phylogenetic tree analysis, bootstrap resampling and reconstruction were carried out 1000 times. These analyses were carried out using Molecular Evolutionary Genetic Analysis (MEGA4) software (Tamura et al., 2007). The gene sequences described in the present study have been deposited in the GenBank database under accession numbers (LC434517–LC434532, LC434537–LC434541, LC430883–LC430888, LC469324–LC469341 and LC469398–LC469603).

### Statistical Analysis

Statistical analysis was performed by the chi-squared test or Fisher’s exact test for categorical variables using SPSS Statistics 17.0 (Advanced Analytics, Inc., Tokyo, Japan). Results were considered statistically significant at *P* < 0.05.

## Results

### Prevalence of RVA

**FIGURE 1:**
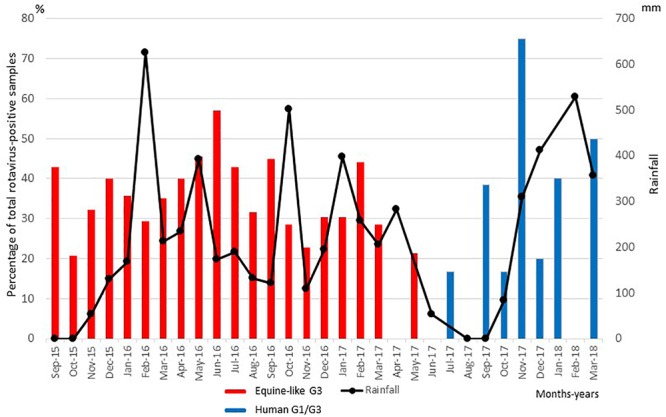
Distribution of equine-like G3P[8]/P[6] (black columns) and global G1/G3P[8]/[6] (gray columns) rotavirus strains collected in East Java, Indonesia, between September 2015 and March 2018. Numbers of rotavirus-positive samples are expressed as percentages of total rotavirus-positive samples in each month of the sampling period.

RVA was detected from 137 samples by immunochromatography testing, and the results were confirmed by PCR. Thus, 31.7% of the 432 pediatric patients with acute diarrhea were found to be infected with RVA. The prevalences of RVA infection in 2015, 2016, and 2017 were 31.6, 36.3, and 24.2%, respectively. The relationship between seasonal distribution of RVA infection and rainfall is shown in Figure 1. RVA infections were seen throughout the year and peaked in November 2017, followed by June 2016. Approximately 88% (121/137) of the RVA-positive samples were from children less than 2 years old. RVA infection was significantly most prevalent in the 6- to 11-month age group (*P* < 0.05).

### Distribution, Transition of G and P Genotypes of RVA and Complete Genome Constellations of RVA Strains

Of the 137 pediatric patients with RVA-associated gastroenteritis, the G genotype was determined in all the cases, while the P genotype was determined in 130 cases. One hundred thirty-seven samples were categorized into three G genotypes (typical human G1, G3, and equine-like G3), and 130 samples were categorized into two P genotypes (P[6] and P[8]). The equine-like G3 strains were determined as the dominant genotype (87.6%), followed by human G1 (9.5%) and human G3 (2.9%). Among the P genotypes identified here, P[8] was the dominant genotype (80.3%), followed by P[6] (14.6%), and 7 strains (5.1%) were non-typeable, because PCR products were not amplified by RT-PCR using 2^nd^ PCR primer set for VP4 genotyping (Tables 1, 2). The most common G-P combination was equine-like G3P[8], accounting for 80.0%, followed by equine-like G3P[6] (12.4%), human G1P[8] (8.8%), G3P[6] (1.5%), G3P[8] (1.5%), and G1P[6] (0.7%) (Table 2). The G-P combinations of the 7 additional samples used as controls in this study were classified into equine-like G3P[8] (collected in 2013) and human G1P[8] (collected in 2013 and 2014). Most of the RVA strains detected were clustered with equine-like G3 during 2015–2017, whereas human genotype G1 became predominant during 2017–2018 (Table 3). In this study, equine-like G3 strains were detected until May 2017, but in July 2017 they were completely replaced by typical human genotypes G1 and G3 (Figure 1).

**Table 2:** Total genotype distribution of RNA strains detected among diarrheal children in East Java during 2015–2018.

RVA genotypes	Number (%) of strains			
	Equine-like G3	Typical human RVA		Total
		G1	G3	
P[6]	17 (12.4%)	1 (0.7%)	2 (1.5%)	20 (14.6%)
P[8]	96 (80.0%)	12 (8.8%)	2 (1.5%)	110 (80.3%)
P[nt]^∗^	7 (5.8%)	0	0	7 (5.1%)
Total	120 (87.6%)	13 (9.5%)	4 (2.9%)	137 (100%)

*^∗^P[nt], non-typeable for the P genotype.*

**Table 3:** Yearly genotype distribution of RVA strains detected among diarrheal children in East Java during 2015–2016.

RVA genotypes/ year	Number (%) of strains			
	Equine-like G3	Typical human RVA		Total
		Human G1	Human G3	
September 2015–August 2016	77 (100.0%)	0	0	77
September 2016–August 2017	43 (97.8%)	1 (2.2%)	0	44
September 2017–March 2018	0	12 (68.8%)	4 (31.2%)	16
Total	120 (87.6%)	13 (9.5%)	54 (2.9%)	137

**FIGURE 2:**
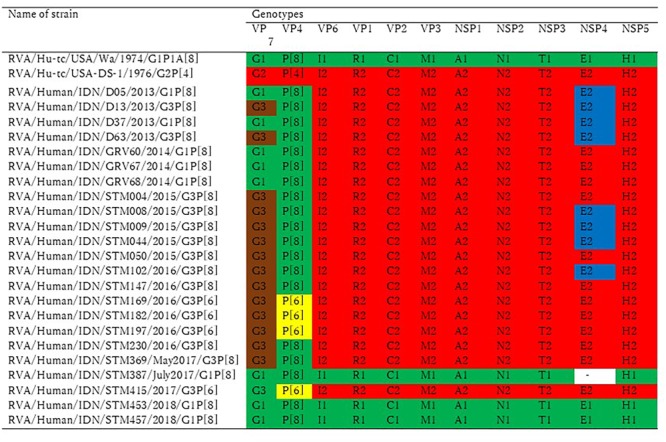
Genotype constellation comparison by the study period. The 23 Indonesian RVA strains in this study were analyzed by whole genome sequences. Green and red indicate Wa-like and DS-1 like gene segments, respectively. The P[6] VP4 genotype is colored yellow, brown is used to indicate a gene segment of equine origin, and blue is used to indicate a gene segment of bovine origin.

The genotype constellation for 11 genome segments of 23 representative strains from 2013 to 2018 was shown in Figure 2. Typical human G1P[8] and equine-like G3P[8] with DS-1-like backbone was exhibited in 2013 and 2014, then epidemic genotype was completely replaced by equine-like G3P[8]/[6] with DS-1-like backbone during 2015–2017 (May). Since July 2017, epidemic genotype has dynamically shifted from equine-like G3P[8]/[6] to typical human G1/G3P[8]/[6] regardless of DS-1-like or Wa-like genotype constellation.

### Nucleotide Sequences and Phylogenetic Analysis of Equine-Like G3 and Other Human RVAs

**FIGURE 3:**
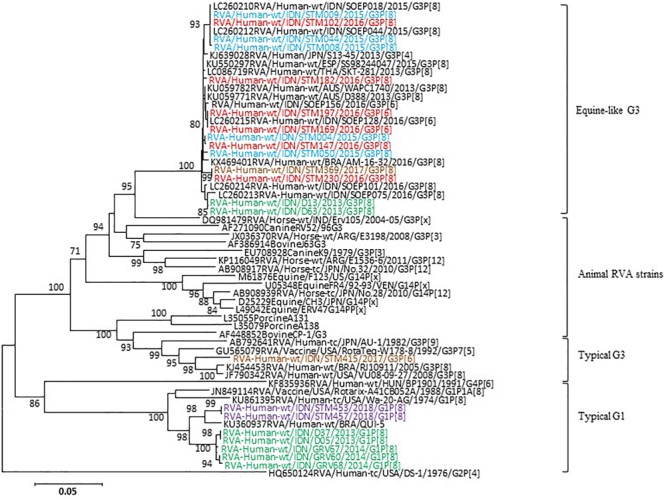
Phylogenetic analysis of RVA VP7 (G genotype) gene. The tree was constructed using the neighbor-joining method. Bootstrap values (>70) are shown at the branch nodes. The names of the RVA strains detected in this study in 2015, 2016, 2017, 2018, and 2013–2014 are highlighted in blue, red, brown, purple, and green, respectively.

**FIGURE 4:**
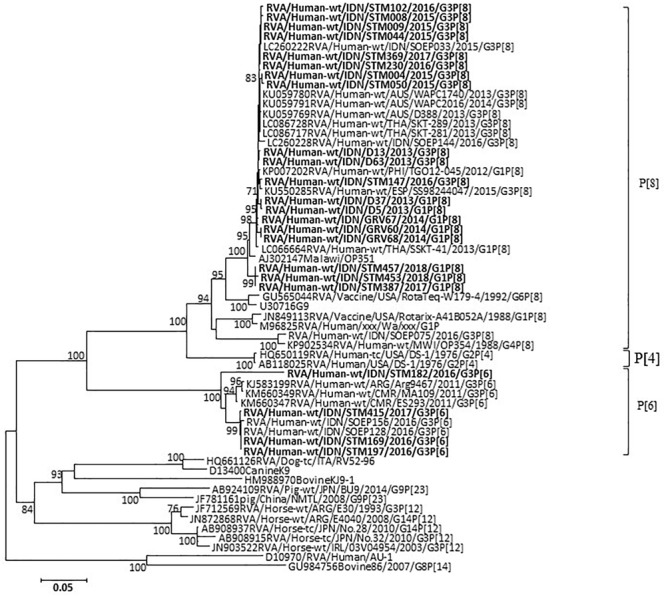
Phylogenetic analysis of RVA VP4 (P genotype) nucleotide sequences. The tree was constructed using the neighbor-joining method. Bootstrap values (>70) are shown at the branch nodes. RVA strains detected in this study are indicated by boldface.

In the VP7 gene, representative strains were classified into equine-like G3 lineage together with the strains previously reported as equine-like G3 in another location in East Java (Utsumi et al., 2018) and in other countries such as Australia, Thailand, Spain, Japan, and Brazil (Malasao et al., 2015; Arana et al., 2016; Cowley et al., 2016; Doro et al., 2016; Guerra et al., 2016; Komoto et al., 2016) (Figure 3). Two control strains collected in 2013 were also classified into equine-like G3 lineage (Figure 3). The rest of the representative strains were classified into human G1 and G3 genotypes together with the strains collected in 2013 and 2014 (Figure 3). The nucleotide sequence identities of the VP7 gene between the equine-like G3 strains in this study were extremely high (99.0–100%) regardless of [P] genotype. Additionally, its identities between equine-like G3 strains in this study and those in other countries were high (98.7–99.6%), suggesting that relationship of equine-like G3P[8] VP7 gene and equine-like G3P[6] VP7 gene were genetically close. In the VP4 gene, the representative strains in this study were classified into P[8] and P[6] genotypes (Figure 4). In the VP4 gene, equine-like G3 and human G1/G3 strains tended to have their own group in P[8] genotype with high nucleotide identities (≥99.1%), respectively. The nucleotide identities between equine-like G3 and human G1/G3 group were 74.8–75.6%. However, RVA strains showed high nucleotide identity (92.5–99.9%) within P[6] genotype regardless of whether equine-like G3 or typical human G1/G3 type. Genotypes determined by phylogenetic analysis were consistent with those determined by RT-PCR.

The phylogenetic tree based on the NSP4 gene showed that eight strains were classified as bovine-like strain and the remaining 15 were classified as human strain (Supplementary Figure S8), while one (STM387) was not amplified. Those bovine-like strains were identified in 2012–2014 in Australia, in 2013 in Japan and in Indonesia (Malasao et al., 2015; Cowley et al., 2016; Utsumi et al., 2018). The representative RVA strains in the VP6, VP1–3, NSP1–NAP3, and NSP5 genes formed a cluster with those of equine-like DS-1-like strains from other countries (Supplementary Figures S1–S9).

### Clinical Data and Severity of RVA Infection

The demographic and major clinical characteristics associated with disease severity are shown in Table 4. The typical symptoms associated with acute viral gastroenteritis are diarrhea, vomiting, fever, and dehydration. The prevalences of vomiting, watery stool, and some dehydration were significantly greater in the RVA-positive children than in the RVA-negative children (Table 4). An increase in the frequency of vomiting (≧5 in 24 h) was highly associated with the RVA-positive children (54.2%), though the difference with the RVA-negative children was not statistically significant (Table 4).

**Table 4:** Clinical features observed among diarrheic children positive for rotavirus infection.

Characteristic	Rotavirus	Rotavirus	*P*-value
	-positive	-negative	
	*N* = 137	*N* = 295	
Male	77 (56.2%)	179 (60.7%)	0.210
**Fever (°C)**		
<37.0	57 (41.6%)	145 (49.2%)	
37.1–38.4	63 (46.0%)	111 (37.6%)	
38.5–38.9	10 (7.3%)	13 (4.4%)	
≧39.0	7 (5.1%)	26 (8.8%)	0.116
**Dehydration**		
Severe	13 (9.5%)	23 (7.8%)	
Some	119 (86.9%)	235 (79.7%)	
No	5 (3.6%)	37 (12.5%)	0.014
Watery stool	113 (82.5%)	211 (71.5%)	0.014
**Duration of diarrhea in days**		
1–4	119 (86.8%)	238 (80.7%)	
5	9 (6.6%)	19 (6.4%)	
≧ 6	9 (6.6%)	38 (12.9%)	0.145
**Maximum number of stools in 24 h**		
1–3	9 (6.6%)	21 (7.1%)	
4–5	32 (23.4%)	76 (25.8%)	
≧ 6	96 (70.0%)	198 (67.1%)	0.828
Presence of vomiting	120 (87.6%)	168 (56.9%)	0.000
**Duration of vomiting in days**		
1	51 (42.5%)	84 (50.0%)	
2	22 (18.3%)	34 (20.2%)	
≧ 3	47 (39.2%)	50 (29.8%)	0.246
**Frequency of vomiting in 24 h**		
1	13 (10.8%)	29 (17.3%)	
2–4	42 (35.0%)	67 (39.9%)	
≧ 5	65 (54.2%)	72 (42.8%)	0.116
**Prognosis**		
Fully recovered/recovered	131 (95.6%)	270 (91.5%)	
Passed away	2 (1.5%)	4 (1.4%)	
Caught any complication	0 (0.0%)	3 (1.0%)	
NA^∗^	4 (3.3%)	13 (4.4%)	0.704

*^∗^NA, no answer.*

We analyzed the demographic and clinical features among children who experienced gastroenteritis with equine-like G3 strains or non-equine-like G3 strains (human G1 and G3). The differences in demographic and clinical characteristics (age, sex, duration, and frequency of diarrhea and vomiting, and dehydration) were not statistically significant between patients with equine-like G3 RVA infection and patients with non-equine-like G3 RVA infection (data not shown). Most of the patients infected with RVA were fully recovered or recovered, regardless of equine-like G3 or typical human G1/G3. There was no statistic differences in prognosis of infants and young children between equine-like and typical human RVA, although the number of samples were too small to compare.

## Discussion

We previously reported that equine-like G3P[8]/[6] DS-1-llike RVA strain was predominant (100%) at a private hospital in the suburbs of Surabaya during 2015–2016 (Utsumi et al., 2018). Here we demonstrated that an unusual RVA genotype (equine-like G3P[8]/[6]) was also the predominant strain in this study population during 2015–2018, who were inpatients at the national referral hospital of East Java located in Surabaya, with decreased prevalence (87.6%). Two of the strains (D13 and D63), collected in 2013 in the same location in East Java and included in this study as controls, were classified into equine-like G3. Our results suggest that equine-like G3 strains, first emerged in Indonesia in 2013 at the same time as the first detection in Australia and Japan, have spread in the eastern part of Java Island, Indonesia. Phylogenetic analysis of the VP7 gene demonstrated that the equine-like G3P[8]/P[6] strains are highly homologous to other strains (99.9–97.3%) from Europe, Asia, South America, and Australia, which several research groups reported as equine-like RVA strains.

The equine-like G3P[8] DS-1 like strain was a rare human/equine reassortant and clustered with equine G3 strains at a high genetic distance by the phylogenetic analysis in VP7 gene and was named as equine-like G3P[8] (Cowley et al., 2016). The equine-like G3 genotype has been found in Australia, Asia, Europe, and South America since 2013, and it has been spreading worldwide, including newly reported cases from Germany (Pietsch and Liebert, 2018) and the United States (Perkins et al., 2017). Equine-like G3P[8]/[6] RVA strains in Indonesia were associated with large numbers of gastroenteritis cases as well as with cases in Australia and Spain (Arana et al., 2016; Cowley et al., 2016). The global emergence of equine-like G3 DS-1-like strains has raised a question whether vaccines have induced selective pressure on zoonotic strains (Luchs et al., 2019). It is important to determine if the global emergence of these novel reassortants may have any implications in RVA vaccine effectiveness.

Typical human G1P[8]/[6] and G3P[8]/[6] strains (12.4%), but not equine-like G3, have been found since July 2017 in the present study. It is noteworthy that the equine-like strains were exclusively detected until May 2017, but in July 2017 they were completely replaced by a typical human RVA (G1 or G3) (Figure 1 and Table 3). Not typical Wa-like strain, but unusual G1P[8] DS-1-like and equine-like G3P[8] DS-1-like strains co-circulated during 2013–2014, whereas equine-like G3P[8]/[6] DS-1-like distinctly circulated during 2015–2017 and G1/G3P[8]/[6] Wa-like/DS-1-like strains were found during July 2017- March 2018 (Figure 2). Now the genotypes has shifted to the typical human G1P[8] and G3P[8], which were previously prevalent in Indonesia. Genotype shift from dominant G1P[8] Wa-like to G2P[4] DS-1-like has been observed in the first 2 years after introduction of Rotarix^®^ (Gurgel et al., 2007; Zeller et al., 2010; Kirkwood et al., 2011; Mukhopadhya et al., 2017). Therefore, it is possible that introduction of RVA vaccine in 2011 in Indonesia resulted in genotype shift in Indonesia. However, there is no plausible explanation for this dynamic transition which occurred only in 2 months. To our knowledge, this is the first report of dynamic changes in rotavirus genotypes from equine-like G3 to typical human G1/G3.

Report from Japan demonstrated G1P[8] DS-1-like was the most prevalent strain during 2012–2014 under vaccination coverage of 39% in 2012–2013 (Fujii et al., 2019a). Another reports from Slovenia demonstrated the most prevalent genotype has shifted from G1P[8] to G4P[8] and G2P[4] under vaccination coverage of 27% in 2007–2013 (Steyer et al., 2014). Our finding also suggests that viral strain change can be influenced by the vaccination even in the country with low vaccine coverage like Indonesia. Further investigation may give us an insight to the direction of the global RVA epidemic in the future.

The overall prevalence of RVA infection in this study was 31.7%, lower than the previously reported data (40%) in East Java in 2013 (Sudarmo et al., 2015). Co-infection with bacteria was suspected in this cohort, since approximately 70% of pediatric patients with rotavirus were found to be co-infected with bacteria in 2013 in this government-owned hospital (Sudarmo et al., 2015). A previous report revealed that the overall prevalence of RVA infection in Java (Central and West), Bali, and West Nusa Tenggara, Indonesia, were 48.7% (2010–2015), 53.7% (2010), 53.3% (2011), 45.1% (2012), 41.7% (2013), 38.7% (2014), and 52.5% (2015) (Mulyani et al., 2018). These results demonstrate a gradual reduction in overall RVA infection by 2014 in those areas. In addition, the prevalence of RVA infection in this study (East Java) was similar to that in Central Java during 2006–2010 (Nirwati et al., 2016). Taken together, our results suggest that vaccination can affect prevalence of RVA infection even in the area with low vaccine coverage. RVA vaccination has not been included in the national immunization program in Indonesia, even though RVA vaccines have been commercially available since 2011 (Mulyani et al., 2018). None of the patients in this study had been immunized with an RVA vaccine. The RVA vaccine coverage in Indonesia is considered rather low since RVA vaccines are too expensive for ordinary citizens (Sudarmo et al., 2015). For this reason, in evaluating RVA vaccine efficacy, careful consideration on the low vaccination coverage should be given.

Introduction of RVA vaccination may be closely associated with the strain selection of RVA genotypes (Roczo-Farkas et al., 2016; Hoque et al., 2018). In addition, in Australia the equine-like RVA strain was found to be more common in areas where Rotarix^®^ vaccine was being administered (Roczo-Farkas et al., 2016). A new study targeting infants with RVA vaccines in private facilities of Surabaya revealed that approximately 70% of infants were immunized with the Rotarix^®^ vaccine (Gunawan et al., 2019). These findings raise the possibility that the Rotarix^®^ vaccine is associated with the emergence of the equine-like strains and that RVA vaccines may induce selective pressure that favors certain genotypes (Roczo-Farkas et al., 2016). The G2P[4] genotype that re-emerged after RVA vaccine introduction in certain areas (Roczo-Farkas et al., 2017; Khandoker et al., 2018) was not found in this study period, but in 2013 it was the predominant genotype in the same location in East Java (Sudarmo et al., 2015).

Infants and children with RVA-associated gastroenteritis exhibited some dehydration, vomiting, and watery diarrhea, which indicate the severity of RVA infection, more frequently than those with non-rotavirus gastroenteritis (Subekti et al., 2002; Mulyani et al., 2018). There were significantly high rates of infection among children younger than 2 years old, especially those between 6 and 11 months old. These findings are well in agreement with those of previous studies (Subekti et al., 2002; Sowmyanarayanan et al., 2012; Gupta et al., 2018). The lower prevalence in older children could be due to acquired immunity through previous exposures (Velazquez, 2009).

In the present study, RVA infection was detected continuously throughout a whole year without any seasonal peak. This was consistent with a study conducted in 2010–2015 in Indonesia (Mulyani et al., 2018) but inconsistent with a study from the same location in 2001–2004, showing peaks in the dry season (June to August) (Wilopo et al., 2009). Other temperate countries typically have RV peaks in winter (dry and cool conditions). These results suggest that considerable attention to local climatic conditions may help our understanding of the prevalence of RVA infection (Levy et al., 2009).

The severity of symptoms in RVA patients with equine-like G3 genotype is similar to that in RVA patients with the typical human RVA G1 and G3 in this study, inconsistent with studies conducted in Australia and Japan, indicating that the equine-like G3P[8] strain was associated with the most episodes of severe gastroenteritis (Cowley et al., 2016; Komoto et al., 2018). Since the number of global strains in this study was limited, it is necessary to accumulate more data on the severity according to each RVA genotype. Additionally, all of the pediatric patients with acute gastroenteritis in this study were hospitalized, suggesting that their symptoms were more severe than those of non-hospitalized cases. Since we failed to collect samples from non-hospitalized children, it is difficult to draw any conclusion about the severity of RVA-associated acute gastroenteritis among children depending on genotype. Further studies involving both hospitalized and non-hospitalized cases of rotavirus gastroenteritis are needed.

Strain diversity occurs over time along with temporal and genetic changes in the viruses (Gupta et al., 2018). The accumulation of knowledge about the features of viral strains may provide insight into what impact the introduction of rotavirus vaccination has on virus epidemiology and circulating genotypes. Thus, continuous monitoring of rotavirus genotypes, especially the emergence of any unusual rotavirus strains, along with data on prevalence and clinical features, will be crucial for assessing the disease burden and for achieving success in future vaccination programs in Indonesia.

## Conclusion

A dynamic change in the rotavirus genotype from equine-like G3 to a typical human genotype (G1 or G3) was observed in this study. Since severe cases of RVA infection were prevalent especially in children aged 6 to 11 months and, more generally, those less than 2 years old, RVA vaccination should be included in Indonesia’s national immunization program. Careful monitoring on the emergence of unusual RVA strains is also needed.

## Ethics Statement

Written informed consent was obtained from parents or guardians of all the children. The study protocol was reviewed and approved by the ethics committees of the government-owned hospital and Airlangga University in Indonesia, and Kobe University in Japan.

## Author Contributions

AA was collected patient’s data. SS, RR, AD, and DR collected samples. TU was responsible for writing the manuscript. RW, ZD, and LY performed all experiments. So, J, and ML collected samples. CM, LD, and TA gave assistance for the research. YD, YF, HS, KK, and IS gave assistance for the research and analysis. IS supervised the study and helped to draft the manuscript. All authors critically revised the manuscript, read and approved the submitted version.

## Conflict of Interest Statement

The authors declare that the research was conducted in the absence of any commercial or financial relationships that could be construed as a potential conflict of interest.

## Footnotes

1 www.data.jma.go.jp/gmd/cpd/monitor/climatview/frame.php

2 http://rotac.regatools.be/
