# Supplementary figures and images for: Molecular Epidemiology and Clinical Features of Rotavirus Infection Among Pediatric Patients in East Java, Indonesia During 2015–2018: Dynamic Changes in Rotavirus Genotypes From Equine-Like G3 to Typical Human G1/G3
Source: Front Microbiol. 2019 May 3;10:940. doi: 10.3389/fmicb.2019.00940 (PMC6510320; doi:10.3389/fmicb.2019.00940)

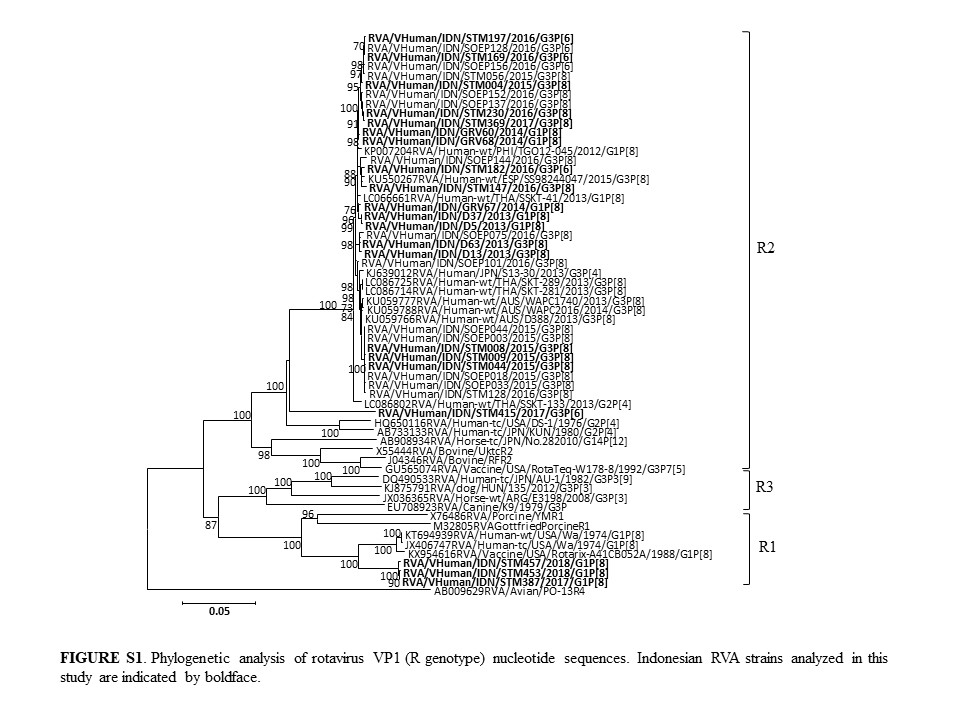

Supplement: Supplementary file 1 [file Image_1.JPEG]

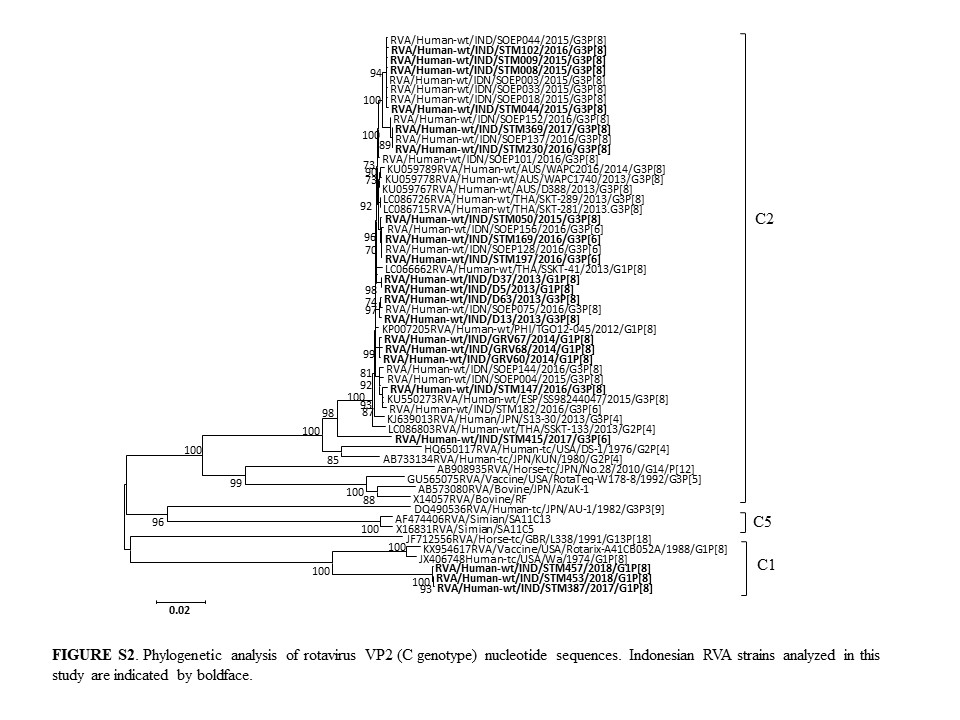

Supplement: Supplementary file 2 [file Image_2.JPEG]

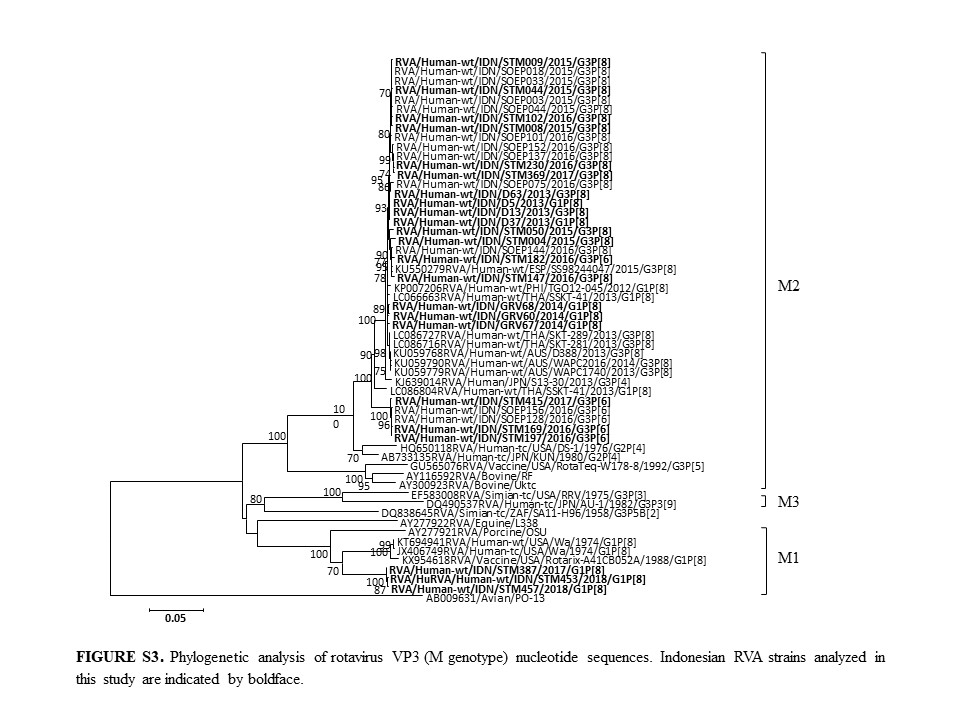

Supplement: Supplementary file 3 [file Image_3.JPEG]

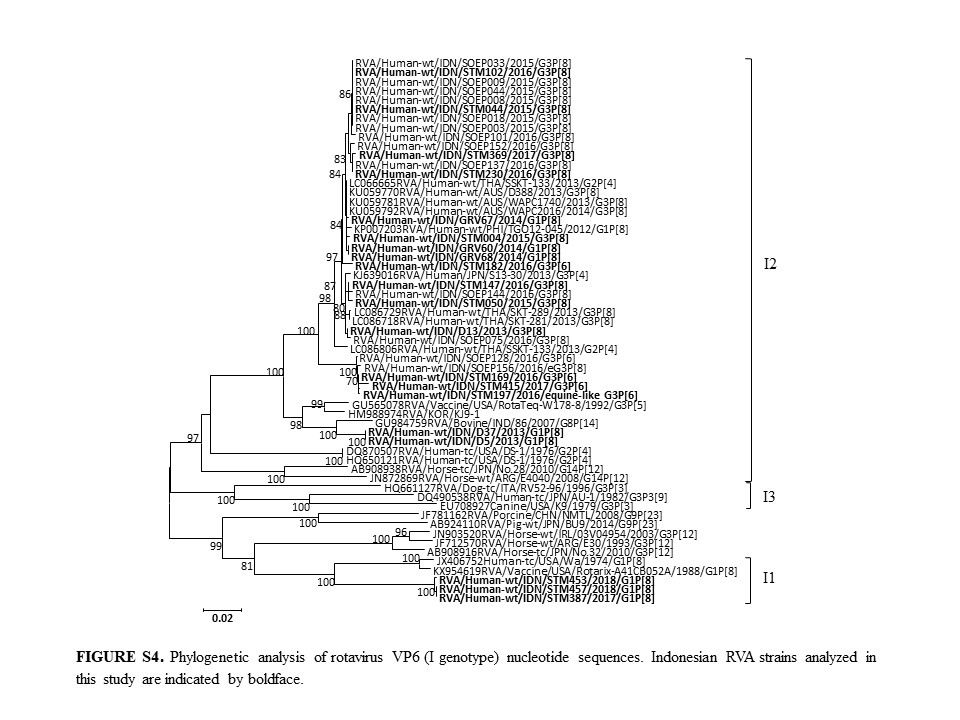

Supplement: Supplementary file 4 [file Image_4.JPEG]

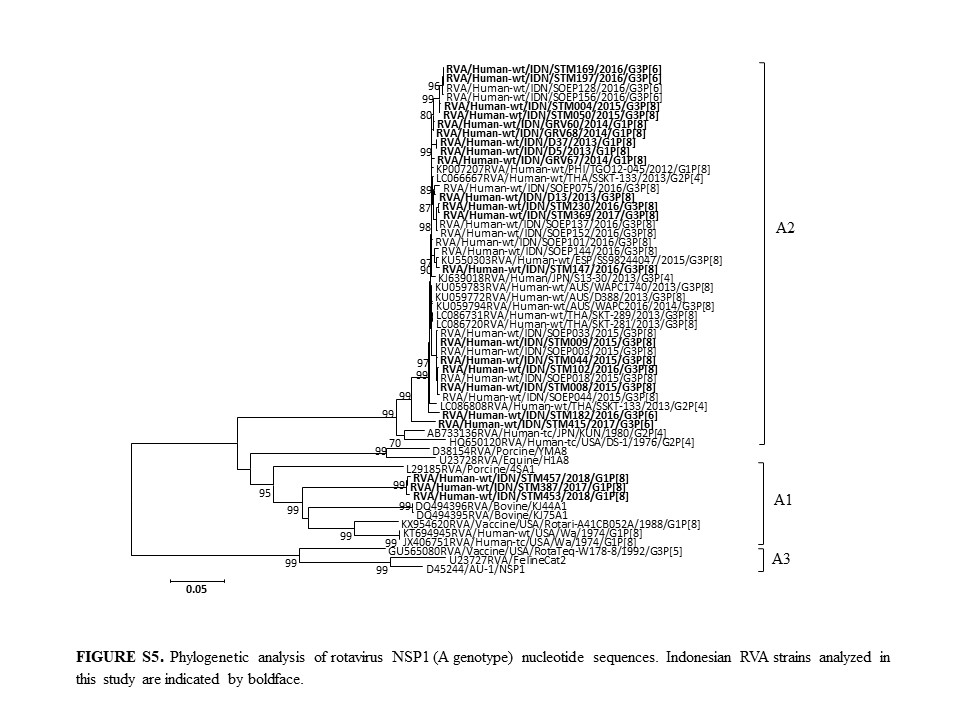

Supplement: Supplementary file 5 [file Image_5.JPEG]

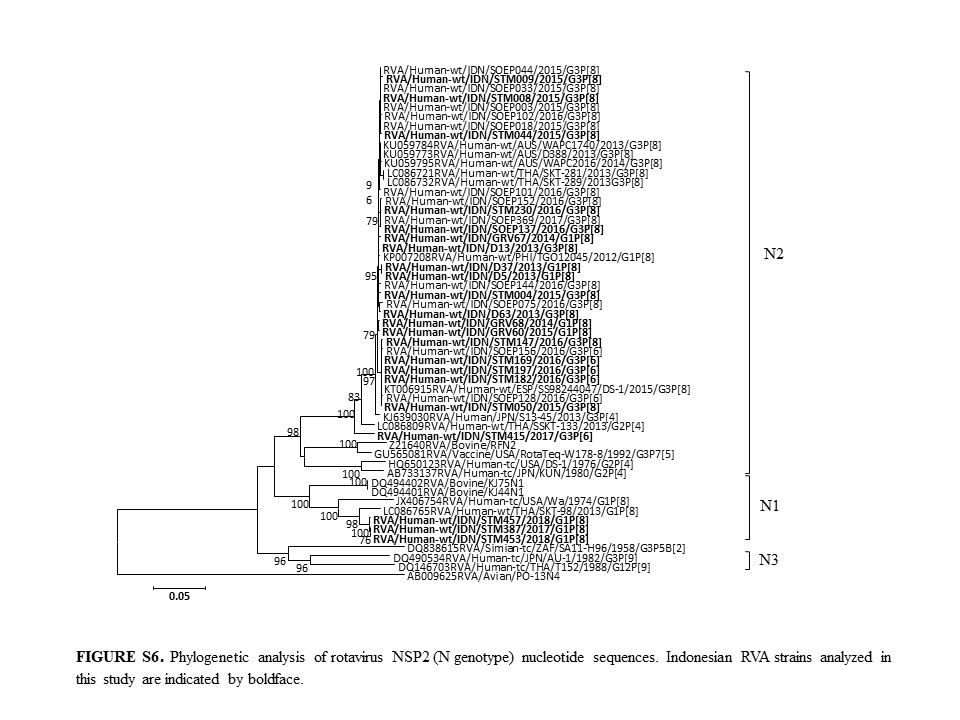

Supplement: Supplementary file 6 [file Image_6.JPEG]

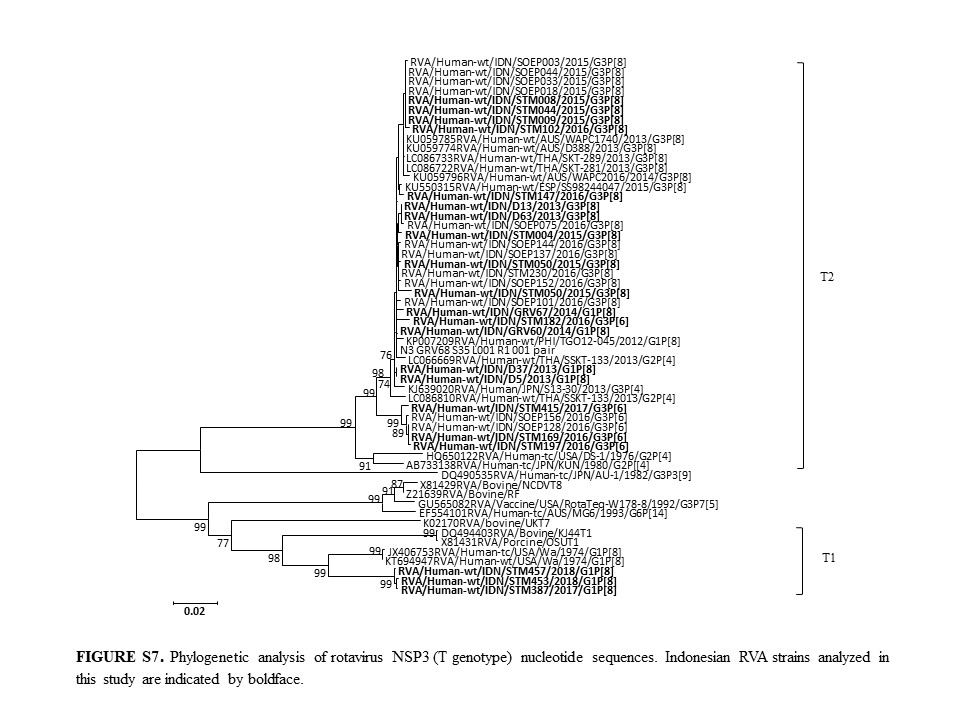

Supplement: Supplementary file 7 [file Image_7.JPEG]

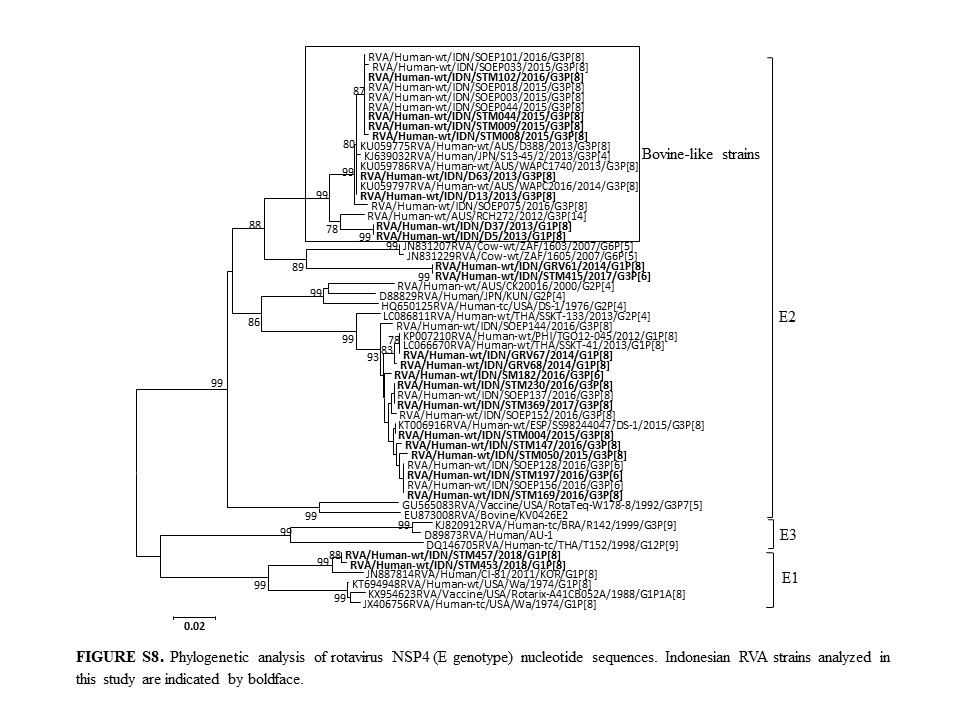

Supplement: Supplementary file 8 [file Image_8.JPEG]

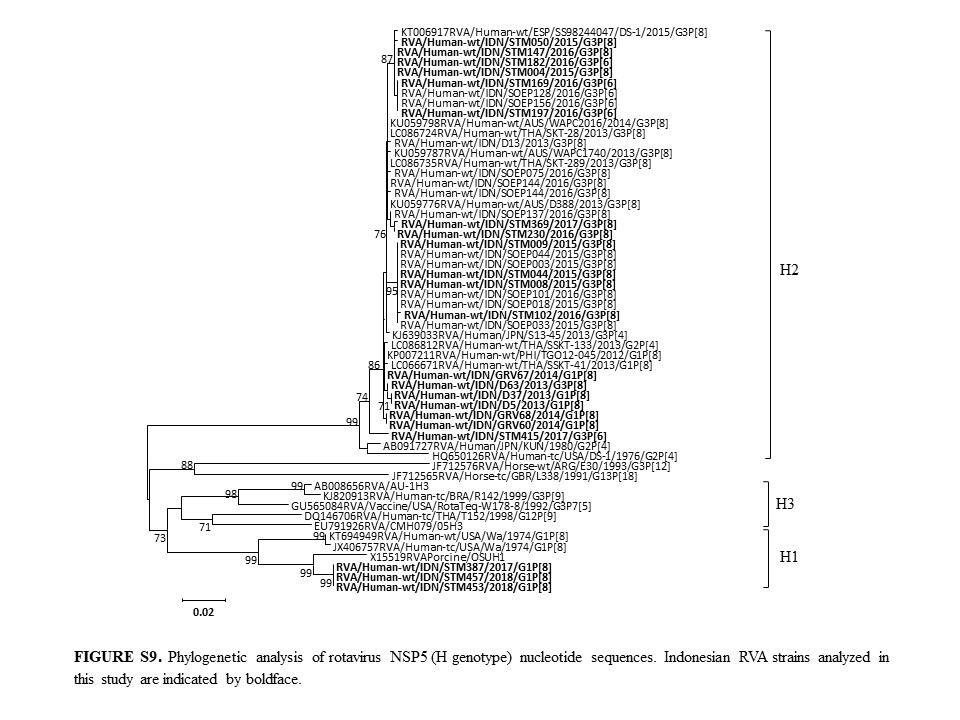

Supplement: Supplementary file 9 [file Image_9.JPEG]
